# Symptom burden and patient characteristics in specialist palliative care: a descriptive analysis

**DOI:** 10.1186/s12904-026-02009-z

**Published:** 2026-02-10

**Authors:** Julia Wikert, Daniela Gesell, Eva Lehmann-Emele, Claudia Bausewein, Maximiliane Jansky, Farina Hodiamont

**Affiliations:** 1 Department of Palliative Medicine, LMU University Hospital, LMU Munich, Marchioninistr. 15, Munich, 81377 Germany; 2 Department of Palliative Medicine, University Medical Center Goettingen, Goettingen, Germany

**Keywords:** Specialist palliative care, Complexity, Symptom burden, Palliative care needs, Functional status

## Abstract

**Background:**

In Germany, specialist palliative care (SPC) is provided by palliative care units (PCU), hospital-based palliative care advisory (PCA) teams, and specialist palliative home care (SPHC) teams. Systematic cross-setting comparisons are lacking. Such comparisons are essential to identify setting-specific patient needs and differences and to inform tailoring of care structures, resource allocation, and training to setting-specific needs. We therefore aim to compare settings regarding patient characteristics, symptom/problem burden, functional status, and palliative care phase.

**Methods:**

Secondary analysis of a prospective, cross-sectional, multi-centre study including 3,115 care episodes (PCU: 753; PCA: 1,568; SPHC: 794) across 29 SPC services. Data collection included symptom/problem burden using the Integrated Palliative care Outcome Scale (IPOS), functional status by Australia-modified Karnofsky Performance Status (AKPS), and Palliative Care Phase. Presence of symptoms was defined as any IPOS item ≥ moderate, sometimes or partly addressed.

**Results:**

Patients had a mean age of 72 years, 51% were female, and 73% had cancer. Weakness (78–90%) and poor mobility (71–85%) were most frequent across all settings. Pain (54%) and poor appetite (63%) were most pronounced in SPHC, while anxiety (61%) dominated in PCA. Median IPOS sum scores were highest in SPHC (24), followed by PCA (22) and PCU (21). Median AKPS scores were overall low, with 30 in inpatient settings and 40 in SPHC, reflecting significant functional impairment. Palliative care phases varied significantly (*p* < .001), with the unstable phase predominant in PCU (43%) and PCA (34%), and the stable phase in SPHC (43%), on admission. High rates of “cannot assess” responses, especially in psychosocial and practical domains, influenced prevalence estimates.

**Conclusions:**

Patients in German SPC experience substantial symptom burden and diverse care needs, varying by setting. The findings highlight the necessity for setting-tailored assessment tools, specialized training, and systemic development to improve care quality and patient outcomes.

## Key message

Patients in SPC experience a consistently high level of care needs, though symptom profiles vary by setting. Each SPC setting plays a vital role in addressing complex care challenges, underscoring the importance of tailored, setting-specific approaches to effectively meet diverse patient needs and improve outcomes across different care environments.

## Introduction

Palliative care aims to address the complex needs of people with life-limiting illnesses by alleviating suffering and improving quality of life for patients and their families throughout the disease trajectory and across care settings [1]. Specialist palliative care (SPC) has evolved internationally to provide comprehensive, multidisciplinary support—including symptom management, psychosocial care, and advance care planning—particularly for patients with high complexity or refractory needs [2–4]. In Germany, SPC is delivered through different settings: Inpatient services include palliative care units (PCU), which are specialised units within hospitals providing intensive symptom management and end-of-life care, as well as palliative care advisory (PCA) teams, which are hospital-based teams supporting non-specialist wards with advice on complex symptom control and care planning. In the community, SPHC teams provide multidisciplinary support to patients at home, coordinating with GPs and families [5, 6]. These services complement generalist palliative care, which is sufficient for most patients but cannot address the full spectrum of complex needs encountered in advanced illness [7].

Previous research has shown that symptom burden and care needs are high among patients receiving SPC, but most studies have either focused on single settings, included only specific diagnosis groups, or have not explicitly compared characteristics between different settings [8–13]. Comprehensive, cross-setting analyses that include all major SPC settings—especially community-based care—are rare in Germany and limited internationally. International studies confirm substantial symptom burden across inpatient and community SPC, but also highlight differences in patient populations, assessment tools, and healthcare structures, which limit direct comparability [14]. Moreover, large-scale registry studies often lack detailed data on psychosocial and practical issues, further restricting holistic comparisons [15–17].

This lack of systematic, comparative data on patients characteristics including symptom and problem burden and functional status across all major SPC settings constrains efforts to tailor care structures and interventions to the specific needs of different patient populations [18]. Understanding both the similarities and differences in patient characteristics and care needs across SPC settings is essential for evidence-based, needs-oriented care planning and resource allocation [19].

This study aims to answer the following research question: How do patient characteristics, symptom burden, functional status, and palliative care phases differ across settings within Germany’s SPC services (PCU, PCA, SPHC).

## Methods

### Study design

This study is a secondary analysis of data collected within the COMPANION project [20], a multicentre, prospective, cross-sectional study aiming to develop a case-mix classification for adult palliative care patients in Germany. The project was funded by the Innovations Fund of the Federal Joint Committee (grant number 01VSF18018) and registered at the German Register for Clinical Studies (DRKS00020517). The reporting of this study follows the STROBE guidelines [21]. Details are reported elsewhere [20].

### Setting and population

Between April 2021 and September 2022, 29 SPC services across Germany (10 PCU, 10 PCA teams, and 9 SPHC teams) consecutively collected data on all newly admitted adult patients (≥ 18 years). Data were collected throughout the entire episode of care, defined as the continuous period from admission to discharge, change of location, or the patient’s death. All data were collected anonymously by healthcare professionals, allowing inclusion regardless of patients’ capacity to provide consent, however, precluding patient-level linkage across episodes due to data protection requirements.

### Data sources and measures

At the beginning of each care episode, sociodemographic data (age, gender, diagnosis) were recorded. Functional status was assessed using the Australia-modified Karnofsky Performance Status (AKPS) [22]. The AKPS is a validated and internationally recognized tool for assessing the functional status of patients with advanced illness, particularly in palliative care using a single rating scale ranging from 100 (full activity, no disease symptoms) to 0 (deceased) in 10-point increments. Symptom and problem burden were measured with the Integrated Palliative care Outcome Scale (IPOS) [23], staff proxy-version. The Integrated Palliative care Outcome Scale (IPOS) is a validated, reliable multidimensional symptom assessment instrument developed for individuals with advanced illness. The scale comprises 17 items, with each item rated on a 5-point scale ranging from 0 to 4, where 0 represents "not at all," 1 indicates "slightly/occasionally," 2 denotes "moderately/sometimes," 3 signifies "severely/most of the time," and 4 corresponds to "overwhelmingly/all the time". The instrument is structured into three distinct subscales: ten items measuring physical symptoms (range 0–40), four items measuring emotional symptoms (range 0–16), and three items measuring communication and practical issues (range 0–12) [23]. The palliative care phase, describing the clinical situation and needs as well as the relevant care plan, was determined by the multidisciplinary team using a culturally adapted German version of the Australian palliative care phase concept [22, 23]. Palliative Care Phases are a validated and widely used clinical tool to classify the care situation into five distinct phases—stable, unstable, deteriorating, terminal, and bereavement—based on a holistic clinical assessment of the patient, their family, and careers.

### Handling of “cannot assess” responses

A central methodological consideration in this study concerns the handling of "cannot assess" (CA) responses in the IPOS assessment. CA responses in IPOS indicate items where symptoms could not be reliably rated by professionals. Our working hypothesis is that professionals, given adequate training and routine, are able to recognize and document relevant symptoms and problems of their patients when present. Accordingly, we consider CA responses as indicative for the absence of the respective symptom or problem (i.e., CA = 0, "not present"). The frequency of CA responses may systematically differ between settings—potentially reflecting not only patient characteristics but also structural or process-related factors. Since we aim to compare different care settings we thereforeexplicitly account for the impact of CA responses in our analyses. To address this, we first conducted a sensitivity analysis comparing two approaches:

a) CA = 0 ("not present"): CA responses are included in the denominator and treated as absence of the symptom/problem vs. b) CA = missing (excluded): CA responses are excluded from the denominator, i.e., only episodes with a valid assessment are considered.

This sensitivity analysis allowed us to quantify the impact of different CA handling strategies on prevalence estimates and setting comparisons. The results of both approaches are reported in the results section, highlighting how prevalence rates and differences between settings change depending on the treatment of CA responses. In line with our working hypothesis and to ensure comparability with previous studies, all subsequent analyses are based on the assumption that CA = 0 ("not present"). This approach ensures representation of all patients in prevalence estimates, avoiding bias from systematic exclusion of patients with communication barriers, cognitive impairment, or limited assessment opportunities. Nevertheless, we acknowledge that high CA rates—particularly for psychosocial and practical items in certain settings—may indicate structural barriers to comprehensive assessment, which are important to consider when interpreting the results.

### Statistical analysis

Descriptive statistics were calculated for all variables, using the first available assessment at the start of each care episode. The presence of symptom and problem burden was defined as any IPOS item rated at least 2 (“moderate”/“sometimes”/“partly addressed”), consistent with previous studies [24, 25]. Episodes with missing values were excluded to allow for calculation of IPOS subscores (physical, emotional, communication/practical domains). Due to non-normal distribution of IPOS subscores and the ordinal nature of AKPS, comparisons between settings were performed using the Kruskal–Wallis H test with Bonferroni-corrected post-hoc pairwise comparisons. Differences in palliative care phases on admission were analysed using Chi-square test of independence. All analyses were conducted using IBM SPSS Statistics version 29. All reported p-values are asymptotic due to the large sample size.

## Results

### Sociodemographic characteristics

Of 3,115 episodes of care 753 (24.2%) originated from PCU (10 teams), 1,568 (50.4%) from PCA (10 teams), and 794 (25.5%) from SPHC (9 teams). Patient characteristics differed significantly across settings: Mean age was 72.4 ± 13.3 years overall, with SPHC patients oldest (75.4 ± 12.3 years) vs. PCA (71.1 ± 13.8) and PCU (72.3 ± 12.9). Pairwise comparisons showed no significant difference between PCA and PCU (p_adj = 0.252), but SPHC patients were significantly older than both PCA (p_adj < 0.001) and PCU patients (p_adj < 0.001). Female patients comprised about 50% overall, with similar distributions across settings (*p* = 0.087). Oncological diagnoses predominated overall (73.1% of 3,097 episodes), but prevalence differed across settings (*p* < 0.001), being highest in PCU (79.3%) vs. PCA (71.1%) and SPHC (71.3%). Care episodes ending in death were most frequent in PCU (58.0%), intermediate in SPHC (51.6%), and lowest in PCA (21.4%) (*p* < 0.001) (see Table 1).

**Table 1:** Clinical characteristics per episode (*n* = 3115)

Setting	Total (*n* = 3,115)	PCU (*n* = 753)	PCA (*n* = 1,568)	SPHC (*n* = 794)
Participating teams	29	10	10	9
Age, years				
Mean ± SD	72.4 ± 13.3	72.3 ± 12.9	71.1 ± 13.8	75.4 ± 12.3
Gender, n (%)				
Female	1,580 (50.7)	402 (53.4)	766 (48.9)	412 (51.9)
Male	1,534 (49.2)	350 (46.5)	802 (51.1)	382 (48.1)
Diverse	0	0	0	0
Missing	1 (0.0)	1 (0.1)	0	0
Length of episode, days				
Median (IQR)	6 (1–196)	9 (1–81)	4 (1–65)	12 (1–196)
Diagnosis, n (%)				
Oncological	2,278 (73.1)	597 (79.3)	1,115 (71.1)	566 (71.3)
Gastrointestinal	612 (19.6)	169 (22.4)	285 (18.2)	158 (19.9)
Respiratory	424 (13.6)	116 (15.4)	253 (16.1)	130 (16.4)
Genitourinary	399 (12.8)	99 (13.1)	178 (11.4)	123 (15.5)
Mamma	203 (6.5)	65 (8.6)	87 (5.5)	51 (6.4)
Melanoma	56 (1.8)	20 (2.7)	56 (3.6)	12 (1.5)
Eye, Head, CNS	85 (2.7)	19 (2.5)	34 (2.2)	12 (1.5)
Lip, mouth, pharynx	56 (1.8)	10 (1.3)	24 (1.5)	10 (1.3)
Endocrinology	25 (0.8)	3 (0.4)	21 (1.3)	1 (0.1)
Others	418 (13.4)	96 (12.7)	177 (11.3)	69 (8.9)
Non-oncological	819 (26.3)	156 (20.7)	444 (28.3)	219 (27.6)
Circulatory	310 (10.0)	69 (9.2)	170 (10.8)	71 (8.9)
Neurology	100 (3.2)	22 (2.9)	41 (2.6)	37 (4.7)
Respiratory	109 (3.5)	15 (2.0)	56 (3.6)	38 (4.8)
Gastrointestinal	64 (2.1)	12 (1.6)	34 (2.2)	18 (2.3)
Genitourinary	54 (1.7)	9 (1.2)	29 (1.8)	16 (2.0)
Mental and behavioural disorders	65 (2.1)	9 (1.2)	23 (1.5)	33 (4.2)
Others	117 (3.8)	20 (2.7)	91 (5.8)	6 (0.8)
Missing	18 (0.6)	0	9 (0.6)	9 (1.1)
Care episode ended with death (%)	1,182 (37.9)	437 (58.0)	335 (21.4)	410 (51.6)

These baseline differences contextualize the setting-specific symptom burden and phase patterns reported below.

### Palliative care phases across settings at admission

**Fig. 1:**
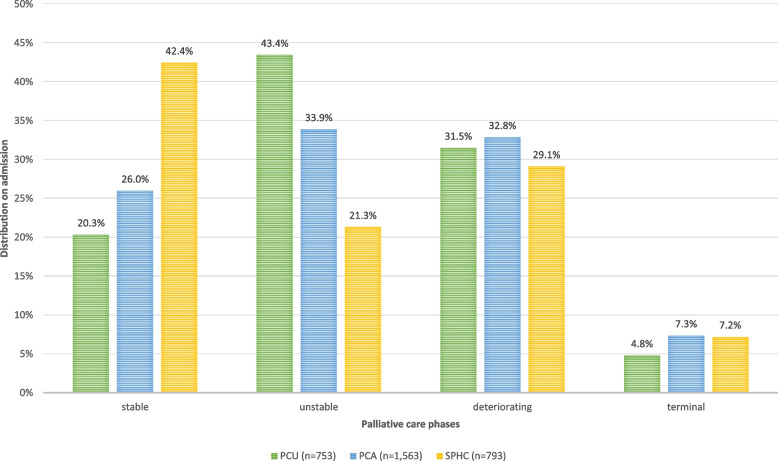
Distribution of palliative care phases across settings on admission

Figure 1 displays the distribution of palliative care phases at the beginning of care episodes. In SPHC, the stable phase was most frequent, accounting for more than 40% of all episodes. In contrast, the unstable phase was (also with more than 40%) most common in PCU. In PCA, the distribution was more balanced, with similar proportions (between 26 and 33%) of episodes beginning in the stable, unstable, and deteriorating phases. The proportion of episodes starting in the deteriorating phase was with about a third (29%−33%), similar across all three settings. The terminal phase was least frequent at episode start in all settings but accounted for a slightly higher share of episodes in PCA and SPHC than in PCU. A chi-square test of independence showed a significant association between setting and palliative care phase, χ^2^(6, *N* = 3115) = 140.49, *p* < 0.001.

### Functional status across settings at admission

**Fig. 2:**
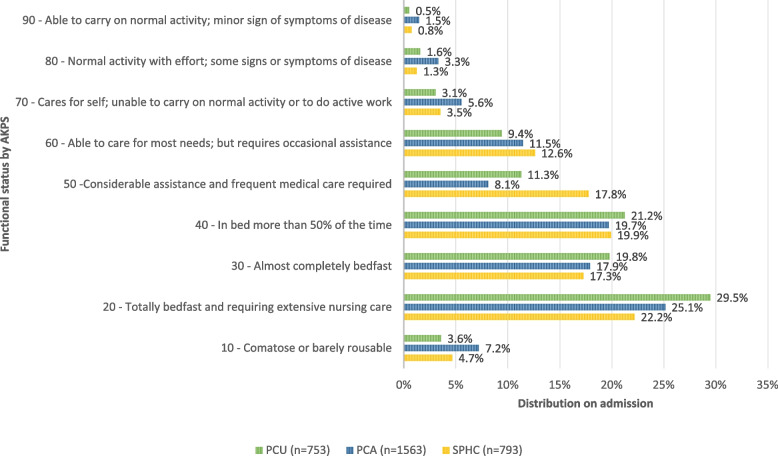
Functional status across settings (PCU, PCA, SPHC)

Functional status, as measured by the AKPS, was severely impaired in the majority of care episodes in all three settings (see Fig. 2). Patients, who were comatouse, bedfast or in bed more than 50% of the time (AKPS between 10–40) were reported at beginning of care in 74.1% of episodes in PCU, 70.0% in PCA, and 64.1% SPHC.

In all three settings, patients were most frequently assessed to be totally bedfast and requiring extensive care (AKPS 20). The largest proportion of totally bedfast patients (AKPS 20; 29.5%) was observed in PCU. PCA showed a slightly higher proportion of comatose or barely arousable patients (AKPS 10; 7.2%) compared to PCU (3.6%) and SPHC (4.7%).

In SPHC, higher proportions of patients were identified in AKPS categories with moderate limitation (AKPS 50–70) compared to the inpatient settings (SPHC: 33.9%, PCU: 23.8%, PCA: 25.1%). Across all settings, only a small minority of patients had AKPS scores indicating normal activity or minor symptoms (AKPS 80–100).

AKPS differed significantly between settings (Kruskal–Wallis test, *p* = 0.003). Median AKPS was 30 in both PCU and PCA, and 40 in SPHC. The most frequent AKPS value (mode) was 20 in all settings. Post-hoc pairwise comparisons indicated a significant difference in AKPS distribution between PCU and SPHC (Bonferroni-adjusted *p* = 0.002), while differences between PCU and PCA, and between PCA and SPHC, were not statistically significant after adjustment (see Table 2).

**Table 2:** Differences in AKPS/IPOS-Scores per setting

	Total	PCU		PCA	SPHC	Kruskal-Wallis-H^d^	df	*p*-value^e^	Post-hoc comparisons^f^				
									Settings	z	*p*-value^e^	n (pair)	Effect size r^g^
AKPS	(*n*=3,109)	(*n*=753)		(*n*=1,563)	(*n*=793)								
Median (range)	40 (10–100)	30 (10–90)		30 (10–100)	40 (10–90)	11.551	2	.003*	PCU - PCA	−1.643	.301	2316	.034
Mode	20	20		20	20				PCU - SPHC	−3.372	.002	1546	.086
IQR	30	30		30	30				PCA - SPHC	−2.264	.071	2356	.047
IPOS													
Total IPOS	*n*=2,920	*n=*734	*n*=1,461		*n*=725	19.264	2	<.001*	PCA-PCU	0,791	.114	2,266	0.02
									PCA-SPHC	−1.660	.000*	2,186	0.04
									PCU-SPHC	−0.869	.147	1,388	0.02
Physical Subscale	*n*=3,051	*n=*751	*n*=1,526		*n*=774	54.422	2	<.001*	PCA-PCU	0.928	.054	2,300	0.02
									PCA-SPHC	−2.864	.000*	2,250	0.06
									PCU-SPHC	−1.936	.000*	1,450	0.05
Emotional Subscale	*n*=3,058	*n=*751	*n*=1,534		*n*=773	7.831	2	.020*	PCA-SPHC	−0.665	.261	2,220	0.01
									PCA-PCU	1.041	.024*	2,285	0.02
									PCU-SPHC	0.376	1.000	1,425	0.01
Communication/Practical issues	*n*=3,004	*n=*738	*n*=1,511		*n*=755	7.154	2	.028*	SPHC-PCU	0.868	0.154	1,464	0.02
									SPHC-PCA	1.004	.026	2,266	0.02
									PCU-PCA	0.136	1.000	2,278	0.00

^d^Group variable: Setting - beginning of care episode

^e^Asymptotic significances are displayed. The significances level is.050

^f^Significance values have been adjusted by the Bonferroni correction for multiple tests

^g^Cohens’ effect size (r =.10 weak, r =.30 middle, r =.50 strong)

### Symptom and problem burden across settings

The presence of each IPOS item across settings is shown in Table 3. Among physical symptoms, burden by weakness (78–90%) and poor mobility (71–85%) was assessed for the vast majority of patient episodes, with the highest prevalence in the home care setting (10%). While the overall prevalence of symptom and problem burden was high in all settings, there were certain symptoms that were more prevalent in the PCU and the SPHC compared to the other settings. In SPHC, the burden by pain (54%), poor appetite (63%), and drowsiness (51%) was reported more frequently than in the inpatient setting. In the PCU, burden by dry mouth (46.2%) was more prevalent than in the other settings. Burden by shortness of breath (35.3%) and vomiting (12.0%) was slightly more prevalent in the PCU than in other settings. The prevalence of physical symptom burden was generally lower in PCA compared to PCU and SPHC, while emotional symptoms such as patient anxiety (61%), depression (56%), and issues related to practical matters (58.0%) were reported most frequently in PCA episodes, with practical matters being approximately 20% more prevalent than in the SPHC setting.

**Table 3:**
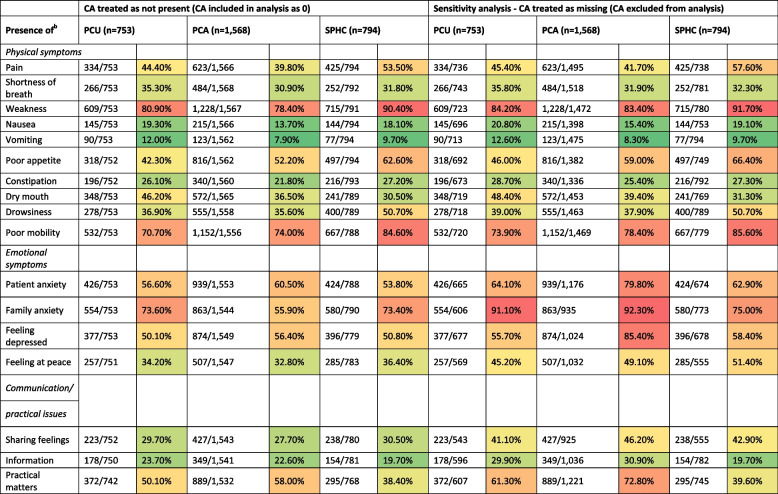
Presence of IPOS items across settings (PCU, PCA, SPHC)

The sensitivity analysis (Table 3) demonstrates that the treatment of ‘cannot assess’ (CA) responses substantially affects prevalence estimates. Across all settings and most items, excluding CA resulted in higher prevalence rates compared to the main analysis. Notably in the PCA setting, prevalences changed clearly depending on the treatment of CA responses. Excluding CA responses increased prevalences of all items, especially for emotional symptoms and communication/practical items such as *family anxiety*, *sharing feelings*, and *practical matters*. CA rates varied widely across items and settings. In episodes of PCA teams the prevalence of *family anxiety* nearly doubled when CA was excluded (55.9% vs. 92.3%).

Table 4 shows the IPOS sum score and IPOS subscores across the three settings. The highest median IPOS sum score was 24 in SPHC, one point higher than in PCU and PCA. The median subscores physical [14] and emotional symptoms [7] were highest in SPHC as well, also with one point difference to the inpatient setting. There were no differences in median subscores of emotional symptoms between inpatient settings and no differences between median subscores of communication/practical issues in all settings.

**Table 4:** IPOS Scores across settings

IPOS	Total (*n* = 3,115)	PCU (*n* = 753)	PCA (*n* = 1,568)	SPHC (*n* = 794)
IPOS sum score, n	2,920	734	1,461	725
Median (min–max)	23 (0–51)	23 (0–48)	23 (0–51)	24 (0–44)
IQR	11	11	12	11
Physical symptoms subscore, n	3,051	751	1,526	774
Median (min–max)	13 (0–33)	13 (0–33)	13 (0–32)	14 (0–33)
IQR	8	8	9	8
Emotional symptoms subscore, n	3,058	751	1,534	773
Median (min–max)	6 (0–16)	6 (0–16)	6 (0–16)	7 (0–16)
IQR	5	5	6	5
Communication/practical Issues subscore, n	3,004	738	1,511	755
Median (min–max)	3 (0–12)	3 (0–12)	3 (0–12)	3 (0–10)
IQR	3	3	4	3

The Kruskal–Wallis H test showed significant differences between settings regarding IPOS scores (sum score and subscores) (Table 4). Mean rank (MRANK) of the IPOS sum score was highest in SPHC (1565.42) as well as the subscore of physical symptoms (1716.89). The mean rank of the emotional subscore was highest in PCU (1591.24), and the mean rank of the communication/practical problems subscore was highest in PCA (1531.08). However, the effect size (r) of post-hoc pairwise comparisons between the settings was small (*r* = 0.00 to 0.06).

## Discussion

This cross-sectional study systematically compared patient characteristics, symptom burden, functional status, and palliative care phases across Germany's major SPC settings (PCU, PCA, SPHC).

Our cohort comprised a high proportion of cancer patients, particularly within inpatient units (approximately 80%), which is in accordance with ongoing reports indicating that oncological diagnoses dominate SPC populations and highlight persistent systemic barriers to the timely referral and integration of non-cancer patients into SPC [16, 26]. These disparities emphasize the necessity for policy initiatives and service developments aimed at removing diagnostic biases and improving access pathways for non-cancer populations.

The distribution of palliative care phases within our cohort, characterized by a predominance of stable patients in home care settings and higher proportions of unstable and terminal patients in hospital-based settings, is consistent with established care trajectories reported in prior studies [27, 28]. This pattern reflects the differing roles SPC settings play within the continuum of care, with ambulatory services frequently providing longer-term support for patients with relatively stable symptoms, while inpatient units more commonly address acute symptom exacerbations and end-of-life care, necessitating intensified resource deployment. Such findings underscore the importance of setting-specific clinical pathways and resource allocation [10]. This distinction also helps to explain the markedly varied proportions of episodes ending in death observed in our data (approximately 58% in PCU, 21% in PCA, and 52% in SPHC). These rates stand in contrast to previous studies from other countries and settings, in which death rates for SPC are usually higher, particularly in inpatient settings and/or end-of-life populations [29, 30]. The comparatively lower death rates in our study very likely reflect the structural impact of episode-level recording. As supported by recent publications, mortality rates are highly sensitive to selection, service structure, and data definitions, which must be acknowledged [31, 32]. Therefore, while our episode-level findings provide valuable insights into service use patterns and care needs, they emphasize the need for harmonized reporting standards and patient-level longitudinal data to improve accurate benchmarking, care planning, and policy decision-making in SPC.

The severe functional impairment observed across all settings consistently underscores the high complexity inherent in SPC populations. The functional status aligns with the distribution of palliative care phases: the predominance of unstable and terminal phases in PCU correlates with the highest proportion of bedfast patients (AKPS 20: 29.5%), while the stable phase predominance in SPHC corresponds to higher proportions of patients with moderate functional limitation (AKPS 50–70: 33.9%). This functional status-palliative care phase interplay reinforces the notion that each SPC setting addresses distinct parts of care during the disease trajectory, e.g. inpatient units managing acute, high-dependency episodes, and home care providing continuity for more stable episodes. These patterns suggest the need for phase-specific and setting-tailored resource models, particularly to enhance community capacity for preventing unnecessary hospitalizations of functionally impaired but stable patients.

Our data notably reveal that practical and psychosocial issues are more pronounced in PCA episodes, which corresponds to the pivotal role of advisory teams as initial points of SPC contact. This aligns with research emphasizing the acute psychosocial distress often experienced by patients and families at the beginning of SPC, highlighting the critical need for comprehensive psychosocial support, family-centered care, and dedicated (time) resources within these teams [33]. Our observation of a consistently high symptom burden, particularly weakness, pain, and psychosocial distress, is in alignment with international literature describing similar symptom profiles across both inpatient and community SPC populations [34–36]. While our study demonstrates a uniformly high symptom burden across all SPC settings, this general statement risks oversimplifying the nuanced differences revealed by our analysis of IPOS subscales and individual symptom prevalence. Although the median scores across the physical, emotional, and communication/practical subscales of IPOS were broadly similar between PCU, PCA, and SPHC, significant differences revealed only small effect sizes, thus lacking clear clinical significance. This suggests that, at the aggregate scale level, the overall burden is comparable; however, the pattern of prevalent symptoms distinctly varies by setting. Such variations are clinically plausible and align with the differing care contexts: symptoms like pain, poor appetite, and drowsiness were more frequently reported in the home care setting, possibly reflecting the impact of managing these symptoms within the domestic environment where patients maintain some degree of normality but are challenged by physical limitations. Conversely, symptoms that may cause hospital admissions, such as severe shortness of breath or vomiting, were relatively more prevalent in inpatient settings. This context-dependent symptom distribution underscores the importance of recognizing setting-specific symptom constellations to optimize resource allocation, care priorities, and patient support. Furthermore, the phenomenon of subjective symptom burden perception may differ particularly in the PCA setting, where patients and families are often newly confronted with the progression of illness and may experience higher levels of distress despite similar symptom severity — explained by the concept of the Calman gap [37]. This psychological phenomenon suggests that perceived symptom burden and coping capacity are influenced by the timing and setting of care delivery, introducing variability in patient-reported outcomes that extends beyond measurable symptom intensity. The prominence of psychosocial and practical problems in PCA further emphasizes the need for enhanced psychosocial expertise, dedicated time, and structural support in these first-contact SPC service. Literature highlights that early phases of PC require sensitive detection and management of emotional distress and practical challenges faced by patients and caregivers, which are crucial for improving quality of life and care satisfaction [38]. Collectively, these insights call for a differentiated interpretation of symptom burden data, where the overall high burden is acknowledged while simultaneously tailoring assessment and intervention strategies to the symptom profiles and psychosocial context characteristic of each SPC setting.

A systematic review on the duration of palliative care before death reported considerable international variability [39] Accordingly, we report shorter lengths of stay than those documented in other studies: In our data, the recorded mean duration of SPHC episodes was 18.4 days (SD 19.3 days; median 6; IQR 3–13), which is notably shorter than the mean length of stay (33.17 days) reported by the recent SAVOIR study (SD 16.66 days; median 30; IQR not stated), a nationwide evaluation of German SPHC structures funded by the German Innovations Fund to assess SPHC outcomes, interactions, and regional differences, which represents more recent findings than the abovementioned review [40]. This discrepancy likely arises because our data possibly include multiple care episodes per individual patient, leading to shorter average episode durations but not necessarily representing shorter overall care periods per patient. Consequently, some patients may appear multiple times within the dataset, which affects the interpretation of episode-level durations and complicates direct comparisons with studies reporting patient-level total care durations. This fragmentation implies that comparisons of care duration across studies require careful consideration of the unit of analysis (episode vs. patient level) as well as study design specifics such as repeated admissions or care transitions.

Methodologically, our study highlights pivotal challenges concerning the handling of CA responses in multidimensional symptom assessment instruments like IPOS. Consistent with calls from Murtagh et al. for improved outcome measurement protocols in palliative care, our sensitivity analyses reveal that handling CA as absence of symptoms can underestimate burden, whereas excluding these responses tends to inflate prevalence estimates and affects between-setting comparisons. These findings underscore the urgent need for transparent, standardized handling of unassessable data to enhance the reliability and validity of symptom surveillance in SPC research and practice. From a quality assurance perspective, high CA rates—particularly in psychosocial and practical domains—should be interpreted as indicators of assessment barriers and potential unmet needs rather than true symptom absence. This underscores the importance of staff training, standardized assessment protocols, and structural measures to ensure comprehensive symptom and problem documentation, especially for vulnerable populations with cognitive impairment or communication difficulties.

### Strengths and limitations

The main strength of this analysis is the inclusion of all patients, regardless of their physical decline, cognitive impairment, or inability to consent. Many earlier studies included only consent-capable or patient-reported data, thus omitting the most severely ill. The present work used proxy assessment to maximize inclusivity, an approach validated but also shown to modestly underestimate emotional and practical burden, particularly in multidimensional instruments such as IPOS. As a general limitation, it should be acknowledged that, although the analyses are based on a comprehensive evidence base, the data do not stem from nationally representative samples of specialist palliative care services in Germany.

Proxy assessments may introduce bias, which likely constrains comparability between settings. A limitation in this context is the possibility that setting-specific differences in symptom interpretation exist; e.g., professionals in home care may rate identical pain burdens as more severe than inpatient staff, depending on their reference standards and perceived ability to intervene. Factors influencing proxy ratings remain unclear and need further investigation. Further, the methodological choices regarding CA coding can be regarded as a limitation, given their substantive impact on conclusions and cross-context comparisons. However, physical symptom prevalence remained relatively stable regardless of CA handling, suggesting robustness in those findings. High CA rates may reflect gaps in assessment processes and unmet needs, and should be seen as an indicator of care quality. Overall, the large sample size, multicentre design, and inclusion of all major SPC settings are clear strengths, providing a comprehensive overview of patient needs.

### Implications for practice

Our findings challenge overly simplistic views of homogeneous symptom burden across SPC and instead emphasize the need for setting-specific awareness. Differences in symptom prevalence and perceived burden across settings require tailored interventions, sufficient psychosocial resources, and staff training to meet the multidimensional needs seen during transitions between care settings. at key care transitions. Moreover, limitations in international comparability due to heterogeneous definitions and measurement tools, especially regarding missing data management, call for harmonized standards for effective benchmarking and quality improvement.

### Implications for research

Future research should focus on developing standardized methodologies for handling unassessable data and capturing nuanced symptom experiences. Longitudinal, patient-focused studies investigating symptom trajectories, coping processes, and transition dynamics will deepen our understanding and inform tailored care. International efforts to harmonize SPC outcome measures are critical to facilitate meaningful comparisons and evidence-based policymaking.

## Conclusion

This study highlights the pervasive symptom burden across German SPC settings, with nuanced differences reflective of specific care contexts. Transparent handling of assessment challenges like CA responses is crucial. Future research should develop standardized protocols for symptom measurement and evaluate interventions tailored to setting-specific needs to improve holistic patient care.

## Data Availability

Data are available upon reasonable request.

## Abbreviations

SPC: Specialist Palliative Care
PCU: Palliative Care Unit
PCA: Palliative Care Advisory
SPHC: Specialist Palliative Home Care
IPOS: Integrated Palliative care Outcome Scale
AKPS: Australia-modified Karnofsky Performance Scale
CA: Cannot assess
